# Multiplex CRISPR/Cas9 system impairs HCMV replication by excising an essential viral gene

**DOI:** 10.1371/journal.pone.0192602

**Published:** 2018-02-15

**Authors:** Janina Gergen, Flora Coulon, Alison Creneguy, Nathan Elain-Duret, Alejandra Gutierrez, Olaf Pinkenburg, Els Verhoeyen, Ignacio Anegon, Tuan Huy Nguyen, Franck Albert Halary, Fabienne Haspot

**Affiliations:** 1 Centre de Recherche en Transplantation et Immunologie UMR 1064, INSERM, Université de Nantes, Nantes, France; 2 Institut de Transplantation Urologie Néphrologie (ITUN), CHU Nantes, Nantes, France; 3 LabEx Immunograft Oncology (LABEX IGO), Nantes, France; 4 CIRI–International Center for Infectiology Research, Team EVIR, Inserm, U1111, Université Claude Bernard Lyon 1, CNRS, UMR5308, Ecole Normale Supérieure de Lyon, Université Lyon, Lyon, France; 5 Institut für medizinische Mikrobiologie, und Krankenhaushygiene, Universität Marburg, Hans-Meerwein-Strasse 2, Marburg, Germany; 6 Inserm, U1065, Centre Méditerranéen de Médecine Moléculaire (C3M), équipe “contrôle métabolique des morts cellulaires”, Nice, France; University of St Andrews, UNITED KINGDOM

## Abstract

Anti-HCMV treatments used in immunosuppressed patients reduce viral replication, but resistant viral strains can emerge. Moreover, these drugs do not target latently infected cells. We designed two anti-viral CRISPR/Cas9 strategies to target the *UL122/123* gene, a key regulator of lytic replication and reactivation from latency. The singleplex strategy contains one gRNA to target the start codon. The multiplex strategy contains three gRNAs to excise the complete *UL122/123* gene. Primary fibroblasts and U-251 MG cells were transduced with lentiviral vectors encoding Cas9 and one or three gRNAs. Both strategies induced mutations in the target gene and a concomitant reduction of immediate early (IE) protein expression in primary fibroblasts. Further detailed analysis in U-251 MG cells showed that the singleplex strategy induced 50% of indels in the viral genome, leading to a reduction in IE protein expression. The multiplex strategy excised the IE gene in 90% of all viral genomes and thus led to the inhibition of IE protein expression. Consequently, viral genome replication and late protein expression were reduced by 90%. Finally, the production of new viral particles was nearly abrogated. In conclusion, the multiplex anti-*UL122/123* CRISPR/Cas9 system can target the viral genome efficiently enough to significantly prevent viral replication.

## Introduction

Human cytomegalovirus (HCMV) primary infection or reactivation can cause severe pathologies in non-immunocompetent individuals[1]^,^[2]. In hematopoietic stem cell transplantation (HSCT), HCMV active replication is the major source of transplant-related morbidity and mortality. Up to one-third of patients with HCMV reactivation develop a CMV disease, with possible end organ diseases[3]^,^[4]. The currently available treatments[5], which target the viral DNA polymerase, are based on nucleotide analogues (Ganciclovir[6] and Cidofovir[7]) and on a non-competitive inhibitor (Foscarnet[8]). The occurrence of Ganciclovir- or Foscarnet-resistant viral strains[9]^,^[10] have urged the development of innovative strategies. Moreover, the current treatments only target the lytic replicating virus and have no impact on the latent viral pool, thus preventing complete virus clearance.

The CRISPR/Cas9 system is an easy, fast and highly potent genome-editing tool. Originally identified as an adaptive immune system in bacteria and archaea against phages and plasmids[11], it is now adapted for use in eukaryotic cells as a suitable two-component system consisting of a Cas9 endonuclease and a chimeric guide RNA (gRNA)[12]. The CRISPR/Cas9 system has also been proposed to be used as an anti-viral strategy to fight latent or chronic viral infections[13–18].

In this study, we hypothesized that disrupting the *UL122/123* gene with a CRISPR/Cas9 system based on one or three gRNAs would prevent viral replication. The *UL122/123* gene encodes several immediate early molecules (IE)[19], which are the first and most essential proteins responsible for the initiation of the viral replication cycle[20]^,^[21]. Indeed, the mutation or shutdown of the *UL122/123* gene leads to a non-replicative virus[22,23]. We induced site-specific cleavage in the *UL122/123* gene using one gRNA and deleted 3300 bp in the *UL122/123* gene from the viral genome using a multiplex strategy with three gRNAs. This led to a strong reduction in IE expression and to the inhibition of late viral protein expression. Overall, the production of new virions was reduced by up to 98% by the multiplex anti-IE CRISPR/Cas9 system. This innovative approach could be used to clear HCMV from infected hematopoietic stem cells before their transplantation into a seronegative recipient.

## Methods

### gRNA design

**Fig 1:**
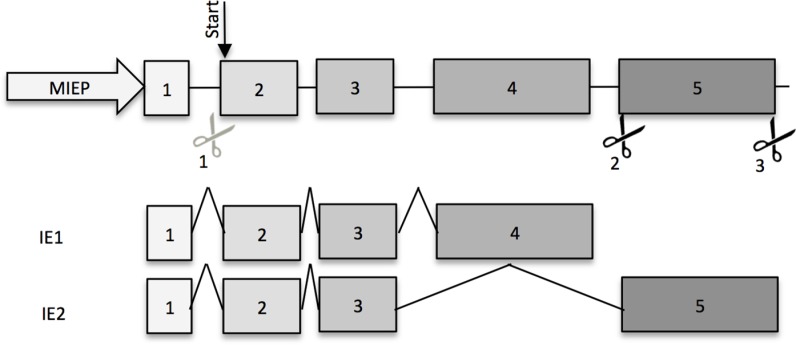
Design of the HCMV-targeting gRNAs. Scheme for the targeted *UL122/123* gene and its major splice variants for IE1 and IE2 with the position of the three designed gRNAs anti-HCMV and their corresponding sequences (Scissors: gRNA/Cas9 complex).

The three gRNAs were designed to target the *UL122/123* gene close to the start codon and at the beginning and end of the exon 5 to target all the IE splice variants (Fig 1). Therefore, the sequence of exons 2 and 5 of the *UL122/123* gene were entered into the CRISPOR software (http://crispor.tefor.net) and the human genome was used as a reference for the calculation of the off-target sites. Fourteen different HCMV genome sequences available at NCBI were aligned for the potential target region to find the conserved regions. gRNAs were selected based on a high selectivity score and low off-target potential assessed by the software and in conserved regions of the viral genome. The following gRNAs were designed: gRNA1, 5’-GGACTCCATCGTGTCAAGGACGG-3’; gRNA2, 5’-GT CCTGGATGGCTGCCTCGATGG-3’; and gRNA3, 5’-GGTGCTACTGGAATCGATACCGG-3’. For the unspecific gRNA, 5’-GAATTTCACCCTGACAAAGGGGG-3’, we used a different DNA virus as the input sequence and chose a gRNA with low off-target potential.

### Cells and virus

MRC5 (primary fibroblasts, RD-Biotech S.A.S, France) and U-251 MG (astrocytoma cell-line formerly known as U-373 MG) cells were cultured in DMEM (Gibco life technology, USA) containing 10% (v/v) FCS (eurobio, France), 100 U/mL penicillin/streptomycin (Gibco life technology), 2 mM L-glutamine (Sigma, USA), and 10 mM HEPES (Gibco life technology), and called complete medium from here on. MRC5 cells were transduced with lentiviral vector (LV) type 1 containing Cas9, a puromycin resistance gene and one or three gRNAs. MRC5 cells were transduced with concentrated LVs diluted at 1:100 in complete medium supplemented with 4 ng/μL polybrene and spinoculated at 1000 × g for 90 min at 33 °C. The inoculum was replaced after 8 h with fresh complete medium. Three days post-transduction, MRC5 cells were treated with 2 μg/mL puromycin for two days (Merck KGaA, Darmstadt, Germany) and subsequently cultured in complete medium. After two weeks, cells were selected a second time with puromycin (0.5 μg/mL) every two days for a period of 9 days and further maintained in complete medium. U-251 MG cells were transduced with LV type 2 containing Cas9-GFP[24] and one or three gRNAs with MOIs ranging from five to ten. After a three-week expanding phase, the transduced cells were FACS-sorted based on their Cas9-GFP levels.

Viral stocks of TB40GFP, Toledo and VR1814 (kindly provided by Dr. Giada Frascaroli and Pr Christian Sinzger, Ulm, Germany) were produced in MRC5 cells infected with an MOI of 0.01 and incubated in a low-FCS (2%) medium. The viral supernatants were harvested between seven and eight days post-infection (pi) and either used directly for infection or purified and concentrated by ultracentrifugation (24000 rpm for 2.5 h at 4 °C) on a 20% sucrose cushion (Qbiogene, USA). The titer of the viral stock solution was determined by FACS analysis of IE-positive MRC5 cells 2 days pi.

### Infection of transduced and control cells

MRC5 cells were plated in a 6-well plate (Falcon, Corning Incorporation, USA) at a density of 1.5x10^5 cells/mL and U-251 MG cells were plated in a 12-well plate (Falcon, Corning Incorporation, USA) at a density of 3x10^5 cells/ml one day prior to the HCMV infection. The cells were subsequently incubated for 2 h with inoculum (MOI 1–0.1) and further cultured in fresh complete medium as defined earlier.

### PCR and T7 assay

Cellular and viral DNA was isolated with the NucleoSpin TriPrep Kit (Macherey-Nagel, Düren, Germany). The *UL122/123* target regions were PCR-amplified by targeting either a small sequence (660 bp amplicon, Primers F: GTTCTCGTTGCAATCCTCGGTCAC and R: CGTGGCGGTAGGGTATGTGT) spanning the IE start codon or a larger PCR sequence consisting of the entire *UL122/123* region (3862 bp amplicon; Primers F: ACATGAGGGGGAGAAGGACA and R: CGTGGCGGTAGGGTATGTGT). For the T7 assay, the small PCR product was purified with a NucleoSpin column. Two hundred nanograms of purified PCR products was denatured for five minutes at 95 °C and slowly re-annealed using three steps consisting of 15 s at 95 °C, 15 s at 85 °C and 30 s at 25 °C, followed by a 30 min digestion at 37 °C with T7 endonuclease (New England Biolab Inc., UK). The reaction was stopped by the addition of 2 μL 0.25 M EDTA, and the samples were analyzed by capillary electrophoresis on a Caliper LabChip GX device (PerkinElmer). The concentration and purity of each band was measured in comparison to an internal marker, which allowed us to quantify our digested and wild type (WT) bands. The percentage of indels was calculated based on the formula from Hsu et al[25].

### TA cloning and sequencing

PCR products from the infected U-251 MG cells four days pi were inserted into an empty ampicillin/kanamycin vector via TA cloning and transformed into competent bacteria with the StrataClone PCR cloning Kit (Agilent Technologie Devision, USA) according to the manufacturer’s instructions. After an overnight incubation at 37 °C, the positive clones were chosen by blue/white selection and sent to MWG (Eurofins Genomics GmbHm Ebersfeld, Germany) for sequencing.

### FACS

The cells were stained with a Live/Dead Fixable Dead Cell Stain Kit (Invitrogen- Thermo Fisher Scientific, USA), fixed in 3.2% PFA for 10 min on ice and then permeabilized with PBS/3% BSA/0.2% Triton on ice for 30 min. Intracellular IE was detected by either an anti-HCMV mAb (clone MAB810R; Millipore, Germany) or an anti-IE/E CMV antibody (Argene Biomérieux, France). HCMV glycoprotein B (gB) was detected intracellularly by a mouse anti-CMV gB antibody (1-M-12, Santa Cruz, USA). An anti-mouse IgG antibody conjugated to Alexa® 647 was used as a secondary antibody in all the staining experiments presented in this study.

### Virion release analysis by trans-infection plaque assay

MRC5 cells were plated in a 24-well plate (Falcon, Corning Incorporation, USA) at a density of 2x10^5 cells/mL to be used the following day for the trans-infection plaque assay. Eight days after HCMV-infection, U-251 MG cells were harvested, counted, serially diluted (from 10^5 to 1 cell per well) and seeded over the MRC5 monolayer cells in duplicate. After an overnight incubation, liquid medium was replaced by 0.8% agarose (Sigma, USA) in MEM. After 7–14 days, the plaques were observed by phase-contrast microscopy and counted.

### Western blot

MRC5 (two days pi) or U-251 MG (eight days pi) cells were harvested and the proteins were extracted using a NucleoSpin TriPrep Kit (Macherey-Nagel, Germany) according to the manufacturer’s protocol. Fifteen microliters of the samples was separated by SDS-PAGE and transferred via semi-dry western blot to a nitrocellulose membrane (GE Healthcare life science, UK) for the U-251 MG cell lysates or via liquid transfer to a PVDF membrane (Millipore) for the MRC5 cell lysates. The membranes were blocked for 2 h in 5% milk in TBST. IE was detected using the mouse anti-CMV antibody (MAB810R, Millipore, Germany) and a donkey anti-mouse HRP antibody (Jackson Immuno Research Labs, USA). After being washed, the membrane was incubated for 5 min with the SuperSignal™ West Pico Chemiluminescent Substrate and the signal was revealed with a Luminescent Image analyzer LAS-4000 (FujiFilm, Japan). Following a short wash in TBST, the antibodies were removed from the membrane with the Restore Western Blot Stripping Buffer Buffer (Thermo Fisher Scientific, USA) for 30 min at room temperature and the membranes were used for subsequent detections. GAPDH or actin detection was used as a housekeeping protein with either mouse anti-GAPDH (6C5, Santa Cruz, USA) or mouse anti-actin (C4, Santa Cruz, USA) antibodies, respectively, and a secondary donkey anti-mouse HRP antibody. The Cas9-GFP from the U-251 MG cell lysate was detected with a rabbit anti-GFP antibody and revealed with a goat anti-rabbit HRP secondary antibody. The Cas9 from the MRC5 cell lysates was detected with an anti-Cas9-A647 antibody (clone 7A9-3A3 Alexa 488, Cell Signaling, The Netherlands) and revealed with a donkey anti-mouse HRP secondary antibody. For the quantification of the proteins on the membrane, the pictures were analyzed with the GIMP 2 software. The membrane background signal was subtracted and the signal intensity of each band was calculated using arbitrary unit/mm^2^. Cas9 expression was normalized to actin expression.

### Statistical analysis

Statistical analysis was performed with the GraphPad Prism software. Kruskal-Wallis test follow by a Dunn post-hoc test was used to compare more than 2 groups was performed to compare the different CRISPR/Cas9 strategies. The Mann-Whitney test was performed to analyze the efficacy of the single strategy over time. *<0.05; **<0.01; ***<0.001; and ****<0.0001.

Please see the supplemental methods in S1 File.

## Results

### Anti-IE CRISPR/Cas9 system reduces IE expression in primary fibroblasts

**Fig 2:**
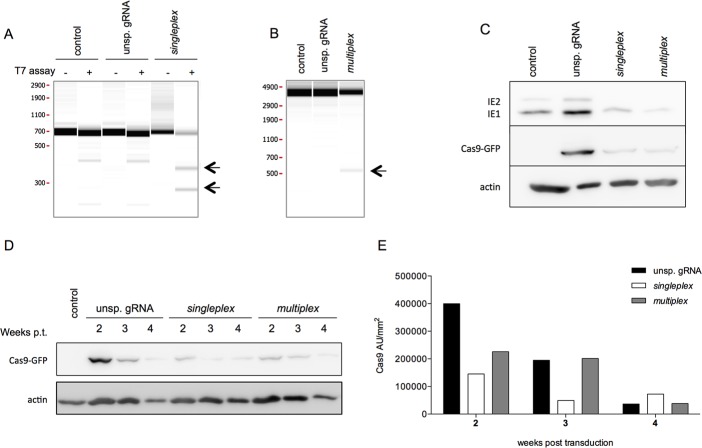
The anti-HCMV CRISPR/Cas9 system induces mutations resulting in a decrease in IE protein expression in primary fibroblasts. MRC5 cells were transduced with one of the three type 1 LVs and selected by puromycin treatment (2 μg/mL) for 2 days. Control (untransduced) and puromycin-resistant MRC5 cells were subcultured prior to infection with Toledo (MOI of 0.1). Two days pi, proteins and DNA were extracted from the infected cells via TriPrep Kit. a) Viral DNA extracts were PCR-amplified at the target region. Amplicons were subsequently subjected to T7 endonuclease to detect the indels induced by the singleplex strategy. b) PCR amplicons of the whole IE gene were analyzed to detect larger deletions induced by the multiplex strategy. The arrows highlight the indels (singleplex) and larger deletions (multiplex) induced by the anti-HCMV CRISPR/Cas9 strategies (one out of three independent experiments is shown). c) Western blot analysis of IE and Cas9 expression 2 days pi (one representative western blot out of 3 independent experiments is shown). d) At each passage, the proteins were extracted with the TriPrep Kit and the Cas9 expression was assessed by Western blot. e) Relative quantification of Cas9 expression based on the Western blot (d) normalized to the housekeeping protein actin. Pt, post-transduction.

To prevent HCMV replication, two anti-HCMV CRISPR/Cas9 strategies based on one (singleplex) or three gRNAs (multiplex) were developed to knockout the *UL122/123* gene that encodes the major immediate early proteins (Fig 1). Each gRNA position was chosen based on a conserved region. These IE proteins are the most essential key regulators of viral replication[20,21]. MRC5 primary fibroblast cells were transduced with LV type 1 (S1 Fig) containing either the anti-HCMV CRISPR/Cas9 system or an unspecific gRNA/Cas9 as an internal control. Cas9-positive cells were selected via puromycin treatment prior to infection with Toledo (MOI 0.1) to assess the effect of the anti-IE strategies on the viral replication. Two days pi, the viral genome was analyzed for indels using a T7-endonuclease assay (Fig 2A). The efficiency of the singleplex cutting was calculated as described elsewhere[26–28] (Table 1). Twenty-nine percent of the viral genome had indels at the target site. To analyze the efficiency of the multiplex strategy, a PCR spanning exons 2 to 5 was performed, which gave a 3862 bp amplicon. Interestingly, while this WT amplicon was strongly detected in the control untransduced MRC5 cells and in MRC5 cells expressing the unsp. gRNA, the multiplex MRC5 cells showed a weaker WT band. Moreover, a weak 500 bp amplicon was also observed (Fig 2B), which probably represents a deletion of 3300 bp between the gRNA1 and gRNA3 target sites. When quantifying the weaker band (500 bp) in comparison with the wild-type band (3862 bp), approximately 5% of the viral genome copies showed this deletion in the *UL122/123* gene. In PCR, the amplification of small products is favored in comparison to longer fragments; thus, these percentages might not represent the exact quantity of mutations in the viral genome extracted from HCMV-infected MRC5 cells. However, the presence of the small PCR fragments is still proof that a part of the viral genome has a larger deletion in the *UL122/123* gene.

**Table 1:** Relative quantification of CRISPR-induced mutations in *UL122/123* gene in MRC5 cells.

	*Mean in %* ±*SD*			
*HCMV strain*	*control*	*unsp*. *gRNA*	singleplex ^*a*^	multiplex ^*b*^
*Toledo*	0 ±0	0 ±0	29.02 ±2.31	5.36 ±0.86

Mean percentage of indels are presented.

^a^ The percentage of mutations for the singleplex strategy is analyzed based on the T7 assay and quantification of the PCR products and cleavage products using a Caliper microfluidics bioanalyzer.

^b^ Larger deletions induced by the multiplex strategy was analyzed by PCR and quantified by a Caliper microfluidics bioanalyzer (n = 2 or 3 independent experiments for the transduced MRC5 cells).

The effect of these mutations in the *UL122/123* gene on IE expression was then analyzed by western blot. The singleplex and multiplex strategies promoted a strong reduction in IE1 expression and nearly abrogated IE2 expression (Fig 2C). The unexpected strong decrease in IE protein expression by the multiplex strategy is probably due to a combination of large deletions (5%) as analyzed by PCR and indels at each target site itself. Furthermore, the cut by the gRNA/Cas9 and the subsequent repair takes at least 5 hours[29], which delays IE expression, even if the gene is correctly repaired.

The analysis of the Cas9 expression by western blot showed that the singleplex and multiplex cells contained only a very low amount of Cas9 compared to the unsp. gRNA strategy. Thus, we questioned the stability of Cas9 expression in the transduced MRC5 cells. As shown in Fig 2D and 2E, Cas9 expression levels were reduced during consecutive subculture steps. After three passages, the Cas9 expression had decreased by 60% (singleplex and multiplex) to 87% (unsp. gRNA). This Cas9 expression was not sufficient to prevent late viral replication events.

Here, we show that anti-HCMV CRISPR/Cas9 strategies disturb the viral genome at the target site, which results in a strong decrease in IE protein expression.

### Stable expression of the anti-IE CRISPR/Cas9 system induces mutations in the *UL122/123* gene in HCMV-infected U-251 MG cells

Since Cas9 expression was not stable in MRC5 cells, we decided to perform a more detailed analysis of the efficiency of the singleplex and multiplex strategies in a HCMV-permissive astrocytoma cell line (U-251 MG). These cells support a full HCMV lytic replication cycle. We designed new LVs expressing a gRNA cassette and Cas9 fused to GFP[24] (S1 Fig). The three type 2 LVs were used to transduce U-251 MG cells at MOIs varying from five to ten., These transduced U-251 MG cells were subsequently FACS sorted based on their Cas9-GFP^high^ expression. All three cell lines had a similar mean of fluorescence (MFI = 1985–2203) for Cas9-GFP expression, which represents a similar expression level of the CRISPR/Cas9 system in the different cell lines (S2 Fig).

**Fig 3:**
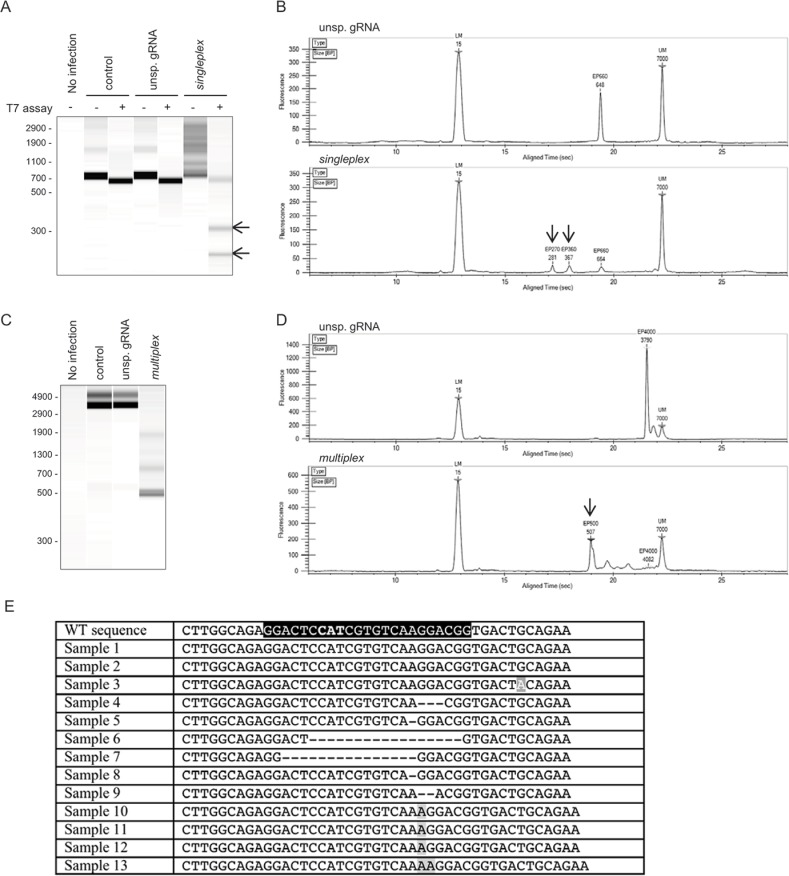
Mutations in the *UL122/123* gene induced by the CRISPR/Cas9 anti-HCMV in U-251 MG cell line. Control and transduced U-251 MG cells were infected with HCMV (Toledo, MOI of 1) and cultured for eight days. Viral DNA was extracted and PCR amplified. a) A T7-assay was performed on the exon 2 PCR amplicon to detect indels induced by the singleplex strategy. b) Electrogram for the T7 assay from the Caliper LabChip analysis for the unsp. gRNA and singleplex strategies. c) Large deletions induced by the multiplex strategy were highlighted by analyzing the whole *UL122/123* gene amplicon. d) Electrogram for the PCR products from the Caliper LabChip analysis identify a major 500 bp amplicon and a smear above with the multiplex strategy. Arrows highlight the indels (singleplex) and larger deletions (multiplex) induced by the anti-HCMV CRISPR/Cas9 strategies. One representative experiment out of three is shown for Toledo, and similar data were found with TB40GFP and VR1814 (n = 3 independent experiments per virus strain). LM, lower marker; UL, upper marker. e) Sequence analysis of the mutations induced by the singleplex strategy in the viral genome four days pi. Black: protospacer + PAM; bold: start codon; gray: insertions; gray-white: substitution.

The three transduced U-251 MG cell lines were infected with three different low passage HCMV strains, including TB40GFP, Toledo and VR1814[30]. Viral genomes were extracted eight days pi to analyze the mutations induced by the gRNA/Cas9 complexes. The efficiencies of the singleplex and multiplex strategies were assessed as described before with the MRC5 cells. The singleplex strategy yielded 30–50% indels (Fig 3A and 3B and Table 2), which was slightly higher than in the MRC5 cells. To confirm that the mutations were induced by the singleplex strategy, the gRNA1 target region was analyzed by Sanger sequencing. Small indels were detected around the gRNA1 cleavage site (Fig 3E). For the multiplex strategy, a 500 bp major amplification product (Fig 3C and 3D) representing the deletion of the target region between gRNA1 and gRNA3 and a smear above representing smaller deletions were detected. The quantification of this band and the smear above in comparison to the WT band revealed that up to 95% of the viral genome was affected by the multiplex strategy (Table 2). Importantly, all three viral strains tested were similarly efficiently targeted, showing the universal usage of our anti-*IE* gRNAs (Table 2). Overall, the multiplex strategy was more efficient than the singleplex strategy and showed significantly higher yields of mutations in the viral genome.

**Table 2:** Relative quantification of CRISPR-induced mutations in *UL122/123* gene in U-251 MG cells.

	*Mean in %* ±*SD*			
*HCMV strain*	*control*	*unsp*. *gRNA*	singleplex ^*a*^	multiplex ^*b*^
*TB40-GFP*	0	0	50.63 ±9.25	95.18 ±5.47
*Toledo*	0.28 ±0.69	0.09 ±0.22	31.18 ±5.18	92.14 ±4.69
*VR1814*	0.86 ±1.62	1.83 ±3.09	46.46 ±11.78	80.00 ±8.58

Mean percentages of indels are presented.

^a^ The efficiency of the singleplex strategy to induce mutations is analyzed based on the T7 assay and quantified by a Caliper microfluidics bioanalyzer.

^b^ Detection of deletions induced by the multiplex strategy is analyzed by PCR and quantified by a Caliper microfluidics bioanalyzer (n = 3 independent experiments per virus strain).

### Dramatic decrease in IE protein expression in HCMV-infected U-251 MG cells expressing gRNA/Cas9

**Fig 4:**
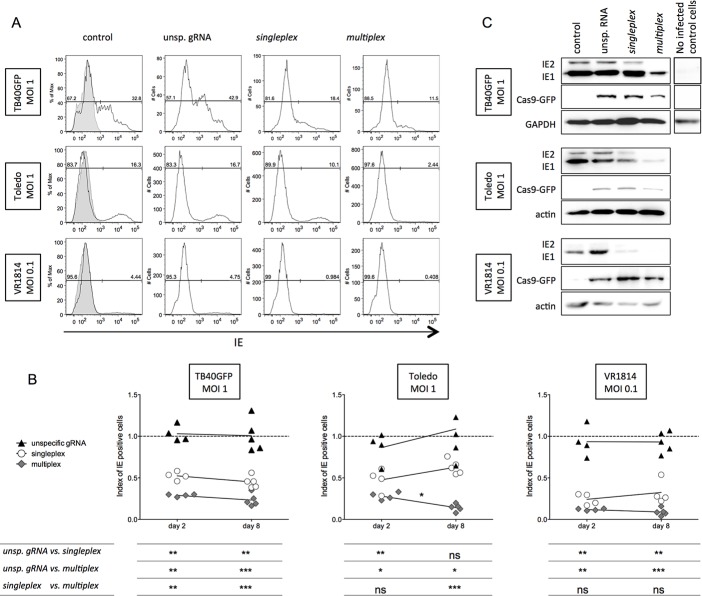
Decrease in IE expression by HCMV-targeting CRISPR/Cas9 systems. Control and transduced U-251 MG cells were infected with HCMV and harvested at two or eight days pi. a) Representative FACS histograms of intranuclear IE expression eight days pi are shown for all U-251 MG cell lines and three different viral strains. The gray histogram represents uninfected U-251 MG cells. b) IE expression in the different U-251 MG cell lines normalized to the HCMV-infected control U-251 MG cells (dashed line) (n = 4 to 5 independent experiments). One-way ANOVA and multiple comparison tests were performed to compare the results within the different cell lines and are presented in the table under each graph. Mann-Whitney tests were performed to analyze each cell line over time (day 2 pi *vs*. day 8 pi). The only significant difference is noted in the graph. c) Western blot analysis of protein extraction obtained using the TriPrep kit eight days pi (one representative western blot out of 3 independent blots is shown for each virus strain as well as for the uninfected control U-251 MG cells).

We analyzed whether the induction of mutations in the IE gene led to a concomitant reduction in IE expression in the different U-251 MG cell lines two and eight days pi with three different viral strains. The unsp. gRNA cell line was equally permissive to HCMV infection compared with the untransduced control cells for the three HCMV strains tested, suggesting that there was no effect of the Cas9/unsp. gRNA on viral infection (Fig 4A and 4B). HCMV-infected singleplex U-251 MG cells showed a reduction in IE-positive cells of up to 50% with TB40GFP or Toledo. The multiplex strategy was significantly more efficient than the singleplex strategy and reduced the amount of IE-positive cells by 75–85% (Fig 4A and 4B). When TB40GFP and Toledo were used at a lower MOI of 0.1, the singleplex and multiplex strategies were even more efficient at controlling IE expression on days two and eight pi (S3 Fig). The endotheliotropic HCMV strain VR1814 could only be used at a low MOI (0.1). Under this condition, the singleplex strategy significantly reduced the IE expression by up to 75% (Fig 4A and 4B). The decrease in IE-positive cells by the multiplex strategy reached up to 95% for VR1814. Comparison of the effects of both strategies between days two and eight pi showed that the decrease in IE expression was stable over time when cells were infected with TB40-GFP. The IE expression decreased significantly over time in cells harboring the multiplex strategy and infected with Toledo (MOI 1) (Fig 4A and 4B). Subsequent analyses for TB40GFP and Toledo were completed with an MOI of 1 to strongly challenge the anti-viral CRISPR/Cas9 system.

Western blot analysis was performed to analyze both major IE splice variants (IE1 and IE2). The expression of both IE variants was impaired by the anti-*UL122/123* CRISPR/Cas9 systems, with greater effects on IE2 than on IE1. Importantly, IE2 expression was undetectable with the multiplex strategy for Toledo and VR1814 (Fig 4C), which could indicate a possible knock-out of IE2. We also analyzed the expression of Cas9 by western blot and could confirm a stable and comparable expression level of Cas9 in all three U-251 MG cell lines. Mutations induced by the singleplex and multiplex strategies led to a significant and stable decrease in the number of IE-positive cells over time and to almost undetectable levels of IE2 protein.

### The multiplex strategy is superior to the singleplex strategy to inhibit viral genome replication and late protein expression

**Fig 5:**
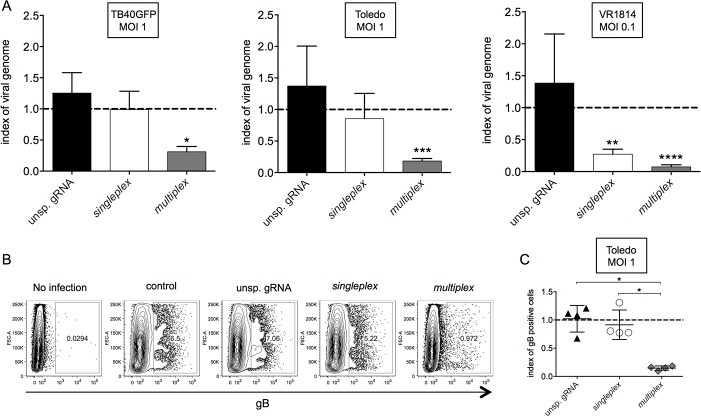
Reduced progression of the viral replication cycle by the multiplex strategy. Transduced and control U-251 MG cells infected with HCMV were harvested eight days pi. a) Relative viral genome quantification normalized to HCMV-infected control U-251 MG cells (dash line) (n = 3 independent experiments, +/- SD). One-way ANOVA and the multiple comparison test were performed and significant differences in the comparison to the control are mentioned. b) Cells were FACS-stained for total gB expression. Representative dot plots of total gB expression after infection with Toledo. c) gB expression normalized to HCMV-infected control U-251 MG cells (dashed line) (triangles: unsp. gRNA; dots: singleplex; diamonds: multiplex) (n = 4 independent experiments). One-way ANOVA and the multiple comparison test were performed.

IE proteins are transactivators and induce the production of delayed early proteins, which are essential for genome replication, for the production of structure proteins, and needed for the assembly of new virions[22,23]. Thus, the disruption of IE expression abrogates the progression of the viral replication cycle. We analyzed the viruses eight days pi; the effects of the anti-*IE* CRISPR/Cas9 strategies on the genome replication were examined via qPCR for the *US8* gene. While the singleplex strategy was only effective for VR1814 at a low MOI, with a decrease of 80% of the viral genome copies, we detected 70 to 90% less viral genome in U-251 MG cells containing the multiplex strategy compared to the untransduced cells for all viral strains (MOI of 1 or 0.1) (Fig 5A).

Furthermore, we analyzed the expression of the viral envelope glycoprotein B (gB), an IE-dependent late viral antigen, by intracellular FACS eight days pi. The untransduced control U-251 MG cells infected with Toledo harbored approximately 6.5% of gB-positive cells (Fig 5B). The use of gRNA1 alone only slightly decreased the percentage of gB-positive cells, while the multiplex strategy nearly abrogated gB expression (Fig 5B and 5C). The gB expression levels for TB40GFP (MOI 1) and VR1814 (MOI 0.1) were not high enough to be detected by FACS analysis.

Overall the progression of the viral replication cycle was dramatically impaired by the multiplex strategy; this is shown by the strong reduction in genome replication and by the decreased expression of the late envelope glycoprotein B.

### The anti-HCMV multiplex strategy strongly impairs virion release from U-251 MG cells

**Fig 6:**
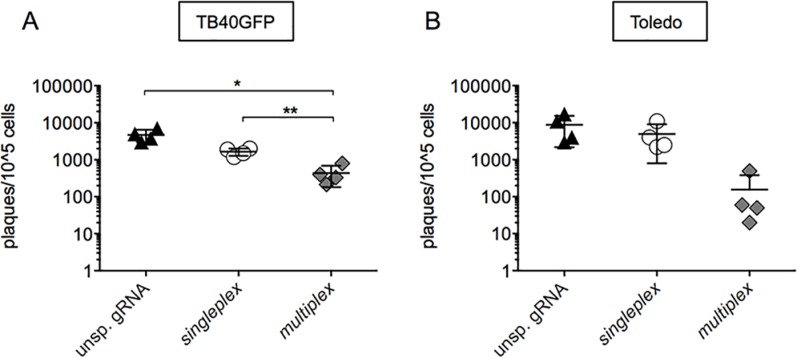
Inhibition of virion release by the CRISPR/Cas9 anti-HCMV. A trans-infection plaque assay was performed on infected U-251 MG cells over MRC5 cells incubated in solid media. Plaque formation was observed (a) 14 days post trans-infection with TB40GFP-infected U-251 MG cell lines or (b) seven days post trans-infection with Toledo-infected U-251 MG cell lines. Each symbol represents the median of duplicates obtained in independent experiments. One-way ANOVA and the multiple comparison test were performed and only significant differences are mentioned in the Figure.

The multiplex anti-*IE* CRISPR/Cas9 system efficiently decreased gB expression and genome replication. To assess the production of infectious viral particles, we established a trans-infection plaque assay based on the direct cell-to-cell transfer of the virus, because U-251 MG cells only poorly secrete HCMV particles into the extracellular space[31]. Control U-251 MG cells and the unsp. gRNA cell line infected with TB40GFP reached a trans-infection plaque-titer of approximately 2500 plaques/10^5 cells (Fig 6A). The singleplex U-251 MG cells released 32% fewer infectious virions (1600 plaques/10^5 cells). Importantly, targeting the *UL122/123* gene with the multiplex strategy decreased virion release by 80% on average (Fig 6A) (436 plaques/10^5 cells). In comparison, Toledo was produced in much higher amounts by the control and unsp. gRNA cells (7287 plaques/10^5 cells) (Fig 6B). The singleplex U-251 MG cells released 67% fewer infectious virions (4925 plaques/10^5 cells) and the multiplex U-251 MG cells showed a remarkable 98% inhibition of virion release (156 plaques/10^5 cells, Fig 6B), though this inhibition was not significantly different.

In conclusion, the multiplex anti-HCMV CRISPR/Cas9 system strongly inhibited the production of infectious viral particles and efficiently prevented viral spreading *in vitro*.

## Discussion

HCMV is a widely spread infection in the human population and can cause severe end organ diseases in immunosuppressed patients, such as solid-organ or HSC transplanted patients. Treatments are effective on replicative viruses and have no effect on the latent virus pool, but resistant strains appear. Here, we propose an alternative antiviral strategy directly targeting the viral genome with CRISPR/Cas9; we excised several exons from the essential *UL122/123* gene and further blocked IE-dependent steps of the viral replication life cycle.

We designed two CRISPR/Cas9 strategies to knock-out the *UL122/123* viral gene based on one or three gRNAs. The *UL122/UL123* gene encodes the Immediate Early molecules, IE1 and IE2, which are the first molecules expressed during the replication cycle and are essentials for the end of latency. Whereas IE2 is known to be essential for viral replication[20,21]^,^[23] and is expressed first during the lytic replication cycle[32], IE1 is responsible for the transcriptional activation of immediate early and delayed early promoters by the inhibition of HDACs [33]^,^[34] and is only essential for infections with a very low MOI[35–37]. Furthermore, the IE molecules are necessary for the initiation of replication from latency and a splice variant of IE1 is essential for viral genome maintenance during latency[38]. The destruction of the *UL122/123* gene would therefore not only be efficient for inhibiting lytic replicating viruses, but would also prevent reactivation from latency and persistence in the host cell. Because IE molecules also influence the host cells in terms of cell cycle regulation and cytokine release[39,40], the inhibition of the expression of those molecules would therefore protect the cells from those side effects. Moreover, Formivirsen, an approved anti-CMV-retinitis drug, is based on an antisense oligonucleotide targeting the *UL122/123* gene that efficiently blocks local HCMV replication[41]. Consequently, the *UL122/123* gene is a suitable target for an anti-HCMV CRISPR/Cas9 system. Targeting the HCMV genome with the CRISPR/Cas9 system has been already investigated by Van Diemen *et al*.[18]. Several gRNAs targeting delayed early genes, which are involved in viral genome replication, were tested using a singleplex approach and achieved short time inhibition of viral replication. Here, we improved the HCMV-targeting by choosing an earlier target gene (immediate early) and three gRNAs for the same gene to block viral protein expression and to prevent further steps in the replication cycle.

In this study, we challenged cells pretreated with the singleplex or multiplex strategy with three different HCMV viral strains. When cells were infected at a low MOI (0.1; for VR1814, Toledo and TB40GFP), the singleplex strategy was efficient at decreasing the expression of IE molecules, as assessed by FACS analysis. As expected, the reduction of IE molecules by the singleplex strategy for the Toledo and TB40GFP viral strains at a higher MOI (1) was not sufficient to prevent viral replication. As observed in the western blot analysis with an MOI of 1, we still have a low amount of IE2 expression in the HCMV-infected singleplex cell line, which was probably sufficient to start the replication cycle [36]. Van Diemen and colleagues also used a singleplex strategy anti-HCMV to target delayed early genes with different efficacies by impairing viral replication, even when a very low MOI (0.05) was used[18]. Furthermore, simultaneously targeting the viral genome with several gRNAs completely abolished the viral cycle as shown so far for HSV-1, HIV and HBV[18,42,43]. In line with these results, we confirmed that targeting HCMV with a multiplex strategy was more efficient than a single gRNA, especially at a high MOI. The multiplex strategy abrogated IE expression at low and high MOIs; this abrogation subsequently led to the blockage of the viral replication cycle, as already mentioned by others [20,22] and shown by us at a high MOI. Importantly, our multiplex strategy was effective on the three viral strains tested, thus opening perspectives for its use in clinical applications.

The use of RNA-guided endonucleases offers advantages over the standard treatments for HCMV infections, which are Ganciclovir and Foscarnet. They block the productive HCMV infection by targeting the viral polymerase UL54[44]. This significantly improves the health of patients facing HCMV diseases, though side effects such as nephrotoxicity and myelosuppression are essential problems for the patient. Developing CRISPR/Cas9 strategies targeting the viral genome with low/no homology to the human genome should be less toxic and have no proven myelosuppressive effects.[15] Moreover, several available high-fidelity Cas9s[45,46] have been shown to significantly reduce off-targets. As previously mentioned, controlling IE protein expression has already been used in the clinic for CMV retinitis in HIV-1 patients before the development of highly active anti-retroviral therapies. The limit of such a strategy involving oligonucleotides is that the effect is only transient and usually does not completely inhibit protein expression. As shown by Hamilton and colleagues[47], the knock-down of HCMV by siRNAs targeting *UL122/123* mRNAs reduces viral replication and virion release. However, the application of siRNAs is very transient and would not prevent HCMV replication over a longer time course. In contrast, the mutations or deletions induced by the CRISPR/Cas9 system are permanent and can provide long-term protection, especially if all viral genome copies are efficiently targeted.

Drug-resistance to Ganciclovir and Foscarnet are due to mutations in the UL97 kinase or UL54 polymerase genes[44]^,^[48]. Escape mutations against the antiviral-CRISPR/Cas9 singleplex system have been shown before with HIV[49,50] and in HCMV[18]. For example, the proposed anti-HCMV CRISPR/Cas9 systems used by Van Diemen *et al*. using one gRNA targeting essential viral genes involved in viral genome replication gave rise to viral escape mutations. Those viral genomes harbored in-frame mutations after being targeted by the anti-viral gRNA/Cas9. The probability of an escape mutation would be significantly lower with a multiplex strategy because the cuts at several targets almost always leads to the deletion of parts or the complete targeted region and not just to small indels. Moreover, two studies on HIV have also shown that the *duplex* strategy can prevent escape mutations and viral breakthrough replication[42,51]. They have proven that the combination of several gRNAs diminishes the probability of in-frame mutations and that a longer exposure to Cas9/gRNAs increases the frequency of larger deletions in the viral genome. Furthermore, a more extensive multiplex strategy was successfully used against EBV, whose 170 kbp genome could be completely destroyed in Raji cells using seven gRNAs simultaneously[13]. Our multiplex strategy is expected to prevent viral escape, which has been described for the other viruses. It mainly induced large deletions (80–95%) after an exposure period of only eight days. Moreover, to reach this goal, high Cas9 expression is needed to target all copies of the viral genome before the expression of the IE molecules, which occurs as early as three hours after HMCV infection[32,52]. It has been shown by Richardson *et al*. that Cas9 remains attached to the DNA for approximately 5.5 h after cleavage and therefore is not available to cut further target sites[29]. During lytic replication, the viral genome copy number increases rapidly and exponentially, and it might not be possible for the Cas9/gRNA complex to target all copies. During natural latency in mononuclear cells from G-CSF-mobilized blood or bone marrow, no more than 13 viral genome copies are present per cell [53]. As a consequence, low Cas9/gRNA expression is expected to target all viral genome copies in a manageable exposure time.

In conclusion, we demonstrated a proof of concept that targeting the *UL122/123* gene in the HCMV genome with a multiplex strategy is efficient to affect viral genome and to inhibit virion release up to 98%. In this study, we showed that even a singleplex strategy is efficient at inhibiting IE expression, if a low MOI is used. The multiplex strategy is superior to the single gRNA at low and high MOIs. Thus, these results pave the way for the development of promising new therapeutic strategies that could be applicable to treat hematopoietic stem cell suspensions. Challenges for such a pre-emptive CRISPR/Cas9 therapy involves an optimized *ex vivo* delivery system, the selection of the targeted cells and the use of high-fidelity Cas9[45,46].

## Supporting information

S1 Fig Constructs of HCMV-targeting CRISPR/Cas9 system. Scheme of the CRISPR/Cas9 constructs including the different gRNA cassettes: unsp. gRNA, singleplex and multiplex and the two lentiviral vectors: type1 with Cas9-T2A-puromycin resistance and type 2 with Cas9-GFP fusion protein. Arrows represent the different promoters. SF: scaffold.
(EPS)

S2 Fig Generation of U-251 MG cell lines expressing the CRISPR/cas9 strategies. U-251 MG cells transduced with one of the three lentiviral vectors type 2 were FACS-serted based on the Cas9-GFP^high^ expression. Post-sort analysis of each U-251 MG cell line is presented. Mean of fluorescence is indicated above each histogram.
(EPS)

S3 Fig Decrease of IE expression by the anti-HCMV CRISPR/Cas9 systems at low MOI. Control and transduced U-251 MG cells were infected with HCMV at an MOI of 0.1 and harvested at two or eight days pi. The different U-251 MG cell lines were stained for intranuclear IE expression and analyzed by FACS. The fractions of IE positive cells were normalized to HCMV-infected control U-251 MG cells (dash line) (n = 3 independent experiments). One-way ANOVA, multiple comparison tests, were performed to compare the results within the different cell lines and are presented in the table under each graph. Mann-Whitney tests were performed to analyze each cell lines over time (day 2 pi *vs* day 8 pi). Only statistical differences are noted in the graph.
(EPS)

S1 File Supplemental methods. Supplemental Methods include the description of the cloning strategies of the lentiviral vectors and the qPCR protocol.
(DOCX)
