# Supplementary material for: Multiplex CRISPR/Cas9 system impairs HCMV replication by excising an essential viral gene
Source: PLoS One. 2018 Feb 15;13(2):e0192602. doi: 10.1371/journal.pone.0192602 (PMC5813945; doi:10.1371/journal.pone.0192602)
Supplement: S1 File — Supplemental Methods include the description of the cloning strategies of the lentiviral vectors and the qPCR protocol. (DOCX) [file pone.0192602.s004.docx]

# Supplemental Material and Methods

## Cloning of the lentiviral vectors

The different gRNAs used in this study were designed with the web tools from *Zhang Lab, MIT 2015* (http://crispr.mit.edu) and the TEFOR/CNRS network (http://tefor.net/crispor/crispor.cgi). Unspecific (unsp.) gRNA 5’GAATTTCACC CTGACAAAGGGGG3’; gRNA1 5’GGACTCCATCGTGTCAAGGACGG3’; gRNA2 5’GT CCTGGATGGCTGCCTCGATGG3’; gRNA3 5’GGTGCTACTGGAATCGATACCGG3’ The gRNA1 and the unsp. gRNA were synthetized as oligonucleotids and cloned into the pX330 (Addgene). Further the gRNA1 or the unsp. gRNA and their promoters were PCR-amplified with the following primers F: 5’ATATGAATTCTT TTGCTCACATGTGAGGGC3’, R: 5’ATATGAATTCCGCGCTAAAAACGGACTAGC3’. The amplicon was then cloned in the LV type 2 expressing the *S. pyrogenes* Cas9-GFP^49^ by EcoRI. The expression cassette encoding the three gRNAs including their own promoters (U6, H1 and 7SK)^50^ for the *multiplex* strategy was synthetized (GeneScript, Piscataway, USA) and cloned by EcoRI in the LV type 2.

The LV Cas9 Puro (Addgene plasmid # 52961) contains the Cas9-P2A-Puromycin as well as a U6 promotor for gRNA expression. The gRNA1 and the unsp. gRNA were synthetized as oligonucleotids and cloned into the LV type 1 expressing the *S. pyrogenes* Cas9 Puro following the Zhang’s Lab protocol. The *mutliplex* strategy was PCR amplified form the LV Cas9-GFP by the following primers: F: TATAttaattaaacgcgtGAGGGCCTATTTCC, R: TATAgaattccgtacgaAAAAAAGCACCGA and cloned into the LV Cas9 Puro via PacI and EcoRI.

## qPCR

Cellular and viral DNA were isolated with the NucleoSpin TriPrep Kit (Macherey-Nagel, Düren, Germany) and the viral genome was quantified by a *US8* qPCR with the following primers F: GGCACCAAATGCAGAGTGAG, R: AAGCCGTATTCCGTTTGCG, and probe: TGGTCCAAGTCCGTGGGCACC (FAM-BHQ-1, Eurofins Genomics GmbH, Ebersberg, Germany). The absolute quantification was performed based on a plasmid standard (10^6 to 10 copies/well) with the TaqMan™ Universal PCR Master Mix (Thermo Fisher Scientific, Waltham, MA USA) at 45 cycles of 94°C 20 s, 57°C 20s and 72°C 20s. Because the percentage of infected cells varied from one experiment to another, the genome copy numbers were first normalized to the amount of total cellular DNA and then expressed as an index of the amount of viral genomes in HCMV-infected untransduced U-251 MG cells.
